# A170 SERUM ALBUMIN AS A MEASURE OF INFLAMMATION OR MALNUTRITION IN INFLAMMATORY BOWEL DISEASE: A CROSS SECTIONAL STUDY

**DOI:** 10.1093/jcag/gwab049.169

**Published:** 2022-02-21

**Authors:** E Squirell, J Fardy, M Borgaonkar, J S McGrath

**Affiliations:** 1 Gastroenterology, Queen’s University, Kingston, ON, Canada; 2 Memorial University of Newfoundland, St. John’s, Canada

## Abstract

**Background:**

Albumin may be both a marker of malnutrition and a marker of inflammation in various disease states but there has been little study of the precise etiology of hypoalbuminemia in patients with Crohn’s disease (CD). Malnutrition, a common complication of active CD, can lead to hypoalbuminemia. Inflammation can also lead to low albumin levels, and previous literature in other inflammatory diseases has suggested that inflammation may be more likely than malnutrition to be a primary driver of hypoalbuminemia.

**Aims:**

In order to investigate the association between serum albumin and disease activity in individuals with Crohn’s disease, we proposed the following research question:

1. In Crohn’s disease, what is the association between serum albumin and two factors – (1) inflammation and (2) malnutrition, and;

2. Is serum albumin an appropriate diagnostic measure of one or both of these factors in patients with CD.

**Methods:**

This study was a single centre cross sectional study of patients with Crohn’s disease in St. John’s, NL. A total of 45 patients with Crohn’s disease were enrolled in the study. Serum albumin was compared with the subjective global assessment (SGA) for nutritional status, the Crohn’s disease activity index (CDAI), and C- Reactive Protein (CRP), a marker of inflammation.

**Results:**

Simple correlation found negative relationships between albumin and inflammation (r = -0.44, p = 0.002) and between albumin and nutritional status (r = -0.406, p = 0.006). Multiple linear regression confirmed independent effect of both inflammation and malnutrition in the model. Participants who were both malnourished and had active inflammation had a mean serum albumin that was 5.32 g/L lower than those who were malnourished but had no active inflammation (p = 0.009) and 4.3 g/L lower than those who had active inflammation but were well-nourished (p = 0.005).

**Conclusions:**

In our study, hypoalbuminemia was independently associated with both malnutrition and inflammation in patients with Crohn’s disease but was most profound in subjects with both malnutrition and active inflammation. These results suggest that although a low serum albumin in Crohn’s disease may be a marker of either malnutrition or active disease, the lowest levels will be seen in patients with both. There are limitations to most surrogate markers of inflammation and malnutrition in inflammatory bowel disease. Serum albumin is a nonspecific marker in this setting but hypoalbuminemia in patients with Crohn’s disease should prompt further investigation with both inflammation and malnutrition in mind.

**Funding Agencies:**

None

